# S Phase Entry of Neural Progenitor Cells Correlates with Increased Blood Flow in the Young Subventricular Zone

**DOI:** 10.1371/journal.pone.0031960

**Published:** 2012-02-16

**Authors:** Benjamin Lacar, Peter Herman, Nathaniel W. Hartman, Fahmeed Hyder, Angelique Bordey

**Affiliations:** 1 Department of Neurosurgery and Cellular and Molecular Physiology, Yale University School of Medicine, New Haven, Connecticut, United States of America; 2 Department of Diagnostic Radiology, Yale University School of Medicine, New Haven, Connecticut, United States of America; 3 Department of Biomedical Engineering, Yale University School of Medicine, New Haven, Connecticut, United States of America; 4 Magnetic Resonance Research Center, Yale University School of Medicine, New Haven, Connecticut, United States of America; 5 Core Center for Quantitative Neuroscience with Magnetic Resonance, Yale University School of Medicine, New Haven, Connecticut, United States of America; University of Udine, Italy

## Abstract

The postnatal subventricular zone (SVZ) contains proliferating neural progenitor cells in close proximity to blood vessels. Insults and drug treatments acutely stimulate cell proliferation in the SVZ, which was assessed by labeling cells entering S phase. Although G1-to-S progression is metabolically demanding on a minute-to-hour time scale, it remains unknown whether increased SVZ cell proliferation is accompanied by a local hemodynamic response. This neurovascular coupling provides energy substrates to active neuronal assemblies. Transcardial dye perfusion revealed the presence of capillaries throughout the SVZ that constrict upon applications of the thromboxane A_2_ receptor agonist U-46119 in acute brain slice preparations. We then monitored *in vivo* blood flow using laser Doppler flowmetry via a microprobe located either in the SVZ or a mature network. U-46119 injections into the lateral ventricle decreased blood flow in the SVZ and the striatum, which are near the ventricle. A 1-hour ventricular injection of epidermal and basic fibroblast growth factor (EGF and bFGF) significantly increased the percentage of Sox2 transcription factor-positive cells in S phase 1.5 hours post-injection. This increase was accompanied by a sustained rise in blood flow in the SVZ but not in the striatum. Direct growth factor injections into the cortex did not alter local blood flow, ruling out direct effects on capillaries. These findings suggest that an acute increase in the number of G1-to-S cycling SVZ cells is accompanied by neurometabolic-vascular coupling, which may provide energy and nutrient for cell cycle progression.

## Introduction

In the adult brain, neuronal activity dictates transfer of oxygen and nutrients from circulation into active neuronal assemblies through a local “neurovascular coupling” process described long ago [1]. Such coupling results in a local hemodynamic (*i.e.* increased blood flow and volume) and metabolic (*i.e.* increased cerebral oxygen consumption) responses in activated regions that form the basis of functional magnetic resonance imaging [2]. The adult brain also contains two neurogenic zones rich in neural progenitor cells (NPCs) and neuroblasts in all animal species examined, including humans [3]–[6]. The largest pool of NPCs is in the subventricular zone (SVZ) along the lateral ventricle that contains a large network of blood vessels [6]–[9]. Although SVZ cells do not generate action potentials, they are active in terms of entering and progressing through the cell cycle, which is a metabolically demanding process ([10], [11] for reviews). In particular, the G1 phase of the cell cycle contains restriction points or checkpoints that are sensitive to growth factor stimulation and nutrient availability necessary for cell growth including DNA replication and protein synthesis (for review see [12]). These latter processes are ATP-dependent and occur on a minute-to-hour time scale [13]–[15].

At any given time, about 15–20% of SVZ cells are actively cycling as assessed by labeling with the nucleoside analog bromodeoxyuridine (BrdU), which is taken up during S phase (e.g. [16], [17]). Nutrients and metabolites supplied through blood perfusion may be sufficient to maintain basal cell proliferation. However, insults (e.g. seizures) and drug treatments acutely increase SVZ cell proliferation as shown by an increase in the percentage of BrdU-positive cells as short as 2–4 hrs post-insult or treatment [18], [19] (for review see [20]). Although such an acute increase in cell proliferation is expected to require increased nutrient availability for G1 and G1-S progression on a minute-to-hour time scale, a correlated hemodynamic response has not been examined.

Here, we examined whether an acute increase in the number of G1-to-S cycling cells in the SVZ is accompanied by a local change in blood flow. We used laser Doppler flowmetry (LDF), which is a well-established method to monitor cerebral blood flow [21] and is very sensitive for even short dynamic blood flow changes [22]. Cell cycle progression and entry into S phase are stimulated by the application of growth factors (EGF and bFGF). EGF and bFGF are well known to increase cell proliferation in the SVZ, and EGF activates the mammalian target of rapamycin (mTOR) pathway known to be necessary for progression through G1 and into S [12], [23]–[27].

## Results

### SVZ capillaries constrict upon U-46119 application in acute slices

**Figure 1:**
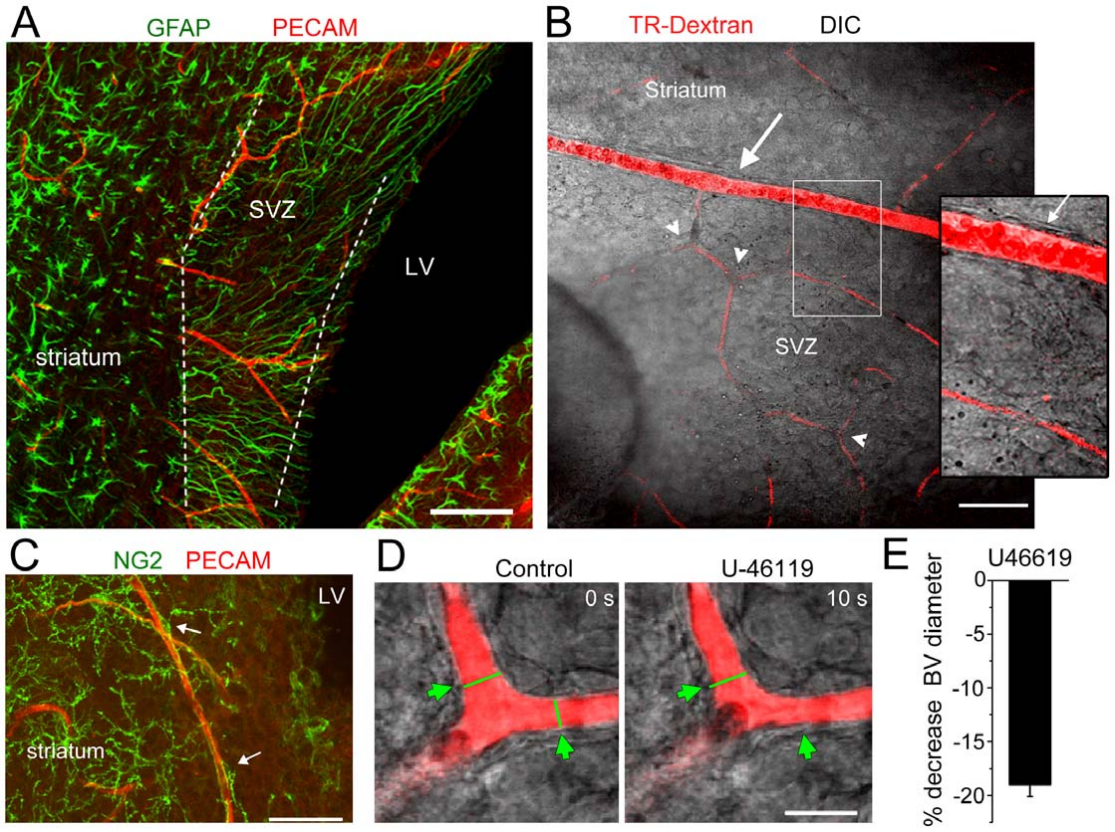
U-46119 constricts SVZ capillaries in acute slices. (**A**) Z-stack projection of GFAP staining (green, mature and SVZ astrocytes) and PECAM (red, blood vessels) in a horizontal section. The dashed lines encompass the SVZ. LV: lateral ventricle. (**B**) Texas Red (TR)-Dextran-filled vessels coursing through the SVZ in a live sagittal section. An arteriole (arrow) branches into capillaries. Note the presence of capillary branchpoints (arrowheads). Inset: zoom of the region delineated by the white rectangle in B. The white arrow points to the smooth muscle cells around the arteriole. (**C**) Z-stack projection of NG2 (green) and PECAM (red) immunofluorescence in a coronal section. NG2 cells on capillaries are pericytes (arrows). (**D**) Image of the capillary before (control) and during U-46119 (100 nM) application. The capillary was loaded with TR-Dextran through cardiac perfusion prior to slicing. The green arrows indicate the sites of constriction. (**E**) Mean % change in blood vessel diameters during and after U-46119 applications. Scale bars: 30 (A), 50 (B), 40 (C), and 15 µm (D).

Blood vessels were visualized by either staining for the endothelial cell marker PECAM in fixed section (**Figure 1A**) or by imaging the fluorescence of transcardially perfused Texas Red dextran (TR-dextran) prior to preparing acute murine slices (**Figure 1B**). Co-immunostaining for PECAM and the astrocytic marker glial fibrillary acidic protein (GFAP) in a horizontal section illustrates a capillary bed throughout the SVZ (**Figure 1A**). In these sections, the SVZ is easily distinguished from the striatum by long GFAP-positive processes that span the SVZ from ventricle to capillaries. In acute sagittal slices, red fluorescent arterioles are visible at the junction between the striatum and the SVZ, which displays lipid droplets in ependymal cells (**Figure 1B**). Arterioles branched into capillaries, which enter the SVZ. Blood vessels were identified as capillaries based on their small diameters (4.6±0.4 µm, n = 30 vessels) and the lack of smooth muscle cells, which are visible on arterioles (white arrow, **Figure 1B**). We next examined whether SVZ capillaries contain pericytes, which are contractile cells and the functional equivalent of smooth muscle cells found on arterioles [28], [29]. We immunostained for the chondroitin sulphate proteoglycan NG2, a marker of pericytes. Although NG2 is also a marker of oligodendrocyte precursor cells (OPC) [30], NG2-positive pericytes have strikingly different morphology from OPCs and lie juxtaposed to capillaries in the SVZ (**Figure 1C**). Finally, to examine whether SVZ capillaries could display a hemodynamic response, we applied the thromboxane A_2_ receptor agonist U-46119 known to constrict capillaries in mature brain networks in acute slices [31]. Capillary diameters were monitored with time-lapse imaging in acute slices from 1 month old mice before, during, and after U-46119 applications. Capillary diameters decreased by 19.0±1.1% (n = 3 capillaries, **Figure 1D and E**). Collectively, these data suggest that the SVZ contains a bed of capillaries that contain the necessary machinery to elicit a hemodynamic response.

### Blood flow measurements *in vivo* using LDF

**Figure 2:**
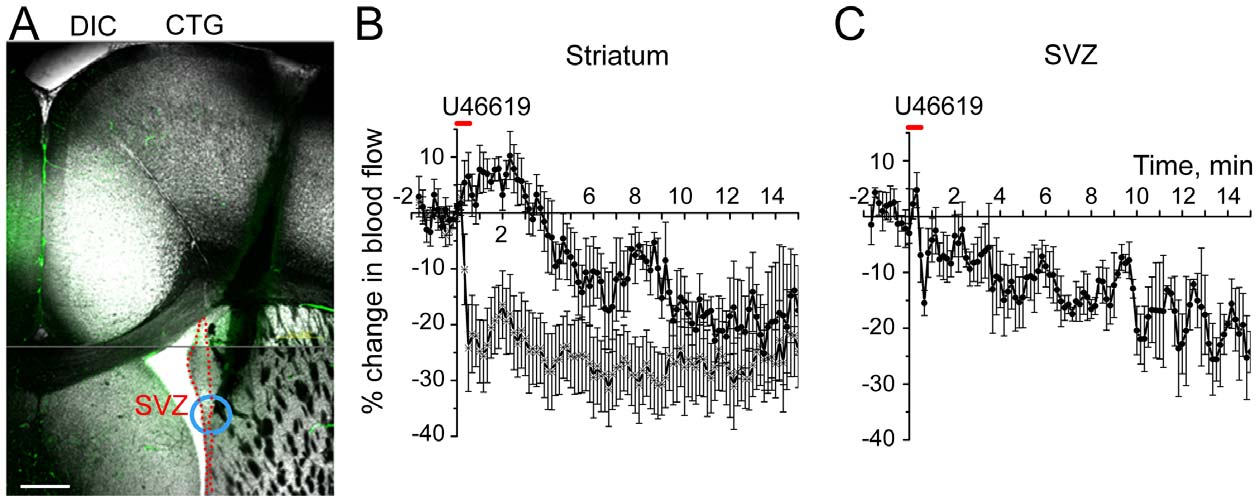
U-46119 decreases blood flow in the SVZ in vivo. (**A**) Image of a coronal section containing the SVZ (delineated by the dashed red line) and the track (black shadow) where the LDF microprobe was located. The blue circle highlights the recorded area. (**B and C**) Mean % change in blood flow (± SEM) against time that were obtained in the striatum (B) and SVZ (C) during and following U-46119 injections (1 µl, 100 nM, 20 s) into the lateral ventricle.

To monitor blood flow *in vivo*, we used a LDF microprobe with a ∼100 µm sampling radius. The tip of a drug- and cell tracker green-filled Hamilton syringe was inserted into the lateral ventricle to inject drug and label the site of drug application. The microprobe was located either in the SVZ (∼50 µm from the lateral ventricle, as shown in **Figure 2A** after removal) or in the striatum to test whether a hemodynamic response could be reliably monitored following U-46119 application in ∼4 month old mice. U-46119 is known to decrease blood flow *in vivo* [32], [33].

Injections of U-46119 (100 nM, 20–30 s) led to a significant 20.8±4.7% decrease in blood flow in the striatum in 9/9 animals (**Figure 2B**). In 4/9 animals, a transient increase in blood flow preceded the progressive decrease, which may be due to a transient dilation of some arterioles as recently shown in slices [32]. U-46119 injections into the lateral ventricle led to a significant 19.6±5.5% decrease in blood flow in the SVZ (n = 3, **Figure 2C**). In each case, the probe location was verified post-recording in serially cut coronal sections (**Figure 2A**). Collectively, these data suggest that blood flow can be monitored and regulated in the SVZ *in vivo*.

### Growth factor infusion induced increases in SVZ cell proliferation and blood flow

To examine whether a change in blood flow was correlated with increased cell proliferation, we applied a combination of two growth factors, EGF and bFGF (each at 0.5 mg/ml) for 30 min in 4–5 weeks old rats. These growth factors are well known to stimulate cell proliferation [24], [25], [27], [34]. In addition, EGF activates the mTOR pathway known to be necessary for progression through G1 and into S, which takes <2 hours according to previous work [23], [26], [35] (for review see [12]).

**Figure 3:**
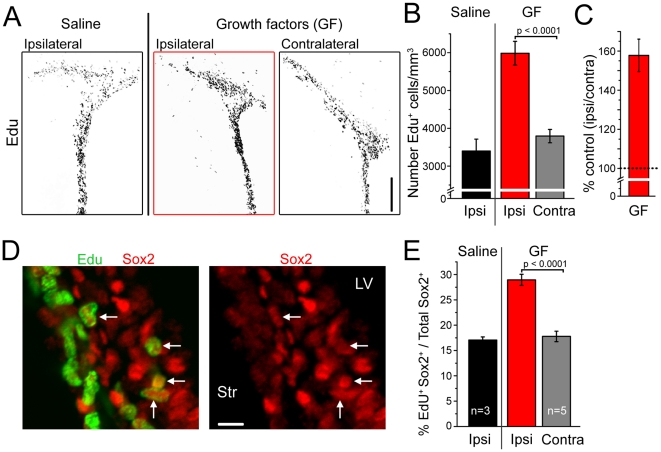
EGF and bFGF increases the number of G1-S cycling SVZ cells that express Sox2. (**A**) Grayscale image of EdU staining in the SVZ in coronal sections from animals that received growth factors (GF) or saline injections. (**B**) Bar graphs of the number of EdU^+^ cells per mm^3^ in the SVZ under different conditions (saline injection or GF injected in the ipsilateral (ipsi) ventricle). (**C**) % of control for the number of EdU^+^ cells in the ipsilateral versus contralateral (contra) SVZ. (**D**) Confocal photographs of EdU (green) and Sox2 (red) immunostaining. Arrows point to double-positive cells in the SVZ. (**E**) Bar graphs of the % of Sox2^+^ cells that were EdU^+^ under different conditions.

To examine the effect of growth factor injection on the number of S phase-entering cells, animals were injected with ethynyl deoxyuridine (EdU) at the end of growth factor injection one hour before the end of the experiments. Like BrdU, EdU is a nucleoside analog of thymidine and is incorporated into DNA during active DNA synthesis (*i.e.* S phase) [36]. There was a significant (60%) increase in the number of cycling cells following growth factor injections in the ipsilateral compared to contralateral SVZ (n = 4 animals, **Figure 3A–C**). The saline-injected ipsilateral and growth-factor injected contralateral exhibited the same density of EdU positive cells (**Figure 3B**). We co-stained for Sox-2, which is preferentially expressed by neural progenitor cells in the SVZ [37], some of which express EGF receptors [38]. We also stained for the neuroblast marker doublecortin (DCX). There was a significant almost 2-fold increase in the percentage of Sox2^+^ cells that were EdU^+^ in the GF-injected side (ipsilateral 29% compared to saline injected or contralateral (both ∼17%) (**Figure 3D–E**). There was no significant change in the percentage of DCX^+^ cells that co-stained for EdU (8.6±1.1% ipsilateral versus 10.2±1.7% contralateral, p = 0.3, n = 4 animals, data not shown).

**Figure 4:**
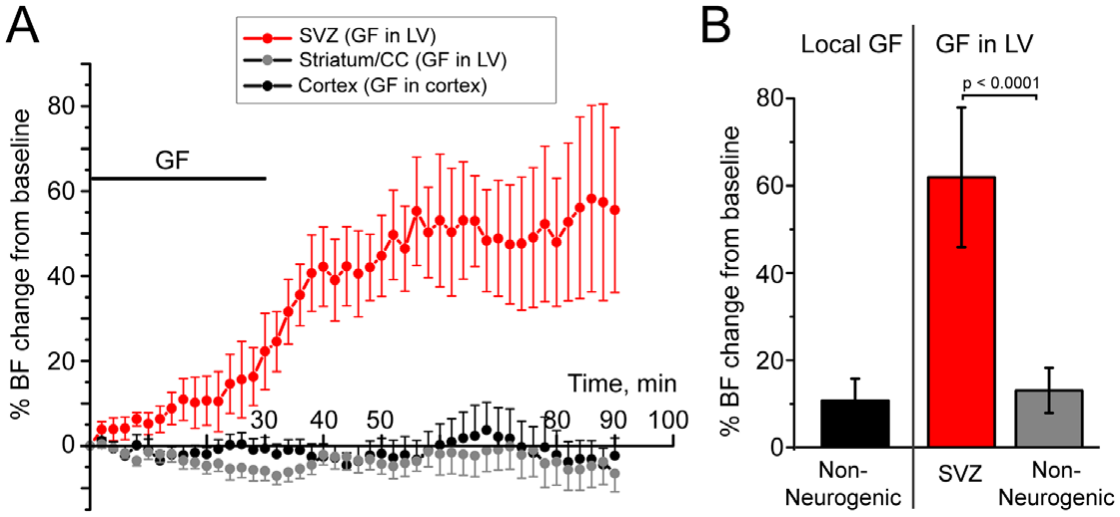
Growth factors increase blood flow selectively in the proliferative neurogenic zone. (**A**) % change in blood flow (BF, ±SEM) from baseline plotted against the recording time during and following growth factor (GF, including EGF+bFGF) injections in the lateral ventricle (LV) or locally where the LDF was recorded (*e.g.* cortex). (**B**) Bar graphs of the maximum % BF change in different regions following GF injections into the LV locally.

The 30 min-injection of growth factors into the lateral ventricle led to a progressive increase in blood flow in the SVZ to a maximum 151±33% of control (n = 4, p<0.0001, red, mean obtained from 60 to 90 min post-infusion, **Figure 4A and B**). The increase reached a plateau at ∼50 min and remained elevated until the end of the recordings (90 min post-infusion). Growth factor injections in the lateral ventricle did not change blood flow in adjacent structures (striatum/corpus callosum, n = 6, grey). Control injections of growth factors into the cortex, which has almost no proliferative cells under normal conditions or following growth factor injection, did not lead to change in blood flow in these regions (n = 9, black, **Figure 4A and B**). These latter experiments rule out a direct effect of growth factors on blood vessels.

## Discussion

Our findings report for the first time that the SVZ contains capillaries with the necessary machinery to increase or decrease vessel diameters, resulting in local blood flow changes that can be monitored *in vivo* using LDF. Most significantly, we found that a growth factor-induced increase in the number of G1-S cycling cells was accompanied by a progressive increase in blood flow suggestive of local neurometabolic-vascular coupling in the SVZ.

Previous studies reported the presence of blood vessels including capillaries in the SVZ [7], [8]. Our study further confirms the presence of capillaries with specialized smooth muscle cells called pericytes in the SVZ. In addition, our data reveal that SVZ capillaries constrict upon application of the thromboxane receptor agonist U-46119 in acute slices, as recently reported in mature neuronal network (cerebellum and striatum) [31]. These findings are consistent with recent studies showing that capillaries have the ability to constrict and dilate through pericytes [28], [29], [31](for review see [39]). Consistent with these data, application of U-46119 *in vivo* reliably decreased blood flow in the SVZ that was monitored using a LDF microprobe. Collectively, time-lapse imaging in acute slices and LDF recordings *in vivo* suggest that the neurogenic SVZ has the proper vascular machinery to locally regulate blood flow.

Injections (30 min) of the growth factors EGF and bFGF induced a relatively rapid increase (in 1–2 hrs) in the number of cells progressing through G1 and entering S phase as shown using labeling for EdU. Such an increase in G1-S cycling cells had been reported following an insult (seizure) and serotoninergic receptor activation [18], [19]. The G1 phase of the cell cycle contains checkpoints where cells stop progressing through the cell cycle (i.e. cell cycle arrest) (for review see [12]). Upon growth factor stimulation and/or nutrient exposure, cells will progress through G1 and enter S, which can take a couple of hours [40](for review see [12]). This checkpoint has been shown to be dependent on activation of mTOR, which is activated by EGF in SVZ cells [23], [26]. Thus, our data suggest that the SVZ contains cells that are arrested in G1 and can quickly progress through a G1 checkpoint and enter S phase upon EGF and bFGF exposure.

Progressing through G1 and entering S phase are ATP-dependent processes that occur on a minute-to-hour time scale [13]–[15]. We thus examined whether the acute growth factor-increased elevation in G1-S cycling cells would be accompanied by a change in blood flow necessary for increased metabolite and nutrient availability. Our data show that growth factor injections were followed by a local increase in blood flow in the SVZ but not in non-neurogenic zones (e.g. cortex or striatum). Nevertheless, it remains to be explored whether SVZ and cortical capillaries could display differential responses to growth factor injections that would contribute to some of the blood flow changes.

Collectively, these data suggest that increased number of G1-S cycling cells in the SVZ is accompanied by a local neurometabolic-vascular coupling. It remains unknown how such coupling occurs in the SVZ. In mature networks, both neurons and astrocytes can participate in such coupling [39]. This will need to be examined in the SVZ, which contains specialized astrocytes with stem cell features that contact capillaries [7], [8], [41], [42].

## Materials and Methods

### Animals

Experiments were performed in 1 month old mice, 25–30 g CD1 mice (∼4 months old), and 55–75 g Sprague-Dawley rats (4–5 weeks old, Charles River Laboratories, MA). Mice were used for experiments with U-46119 applications in slices and *in vivo*. The growth factor experiments were all performed in rats.

### Ethics Statement

Protocols were approved by the Yale University Institutional Animal Care and Use Committee.

### Immunostaining and 5-ethynyl-2′-deoxyuridine (EdU) experiments

Immunostaining was performed in free-floating 100 µm-thick slices as described [43]. The primary antibodies include: rabbit anti-GFAP (1∶1000, Dako), rat anti-PECAM (1∶100, BD Biosciences), rabbit anti-NG2 (1∶200, Chemicon), and rabbit anti-Sox2 (1∶200, Abcam). Z-section images were acquired on a confocal microscope (FluoView 1000) with a 20× dry objective (N.A. 0.75), analyzed using Imaris 4.0 (Bitplane AG) and reconstructed in Photoshop CS3. Staining for EdU positive cells was performed using the Click-iT EdU Cell proliferation assay (Invitrogen) followed by Sox2 immunostaining.

### Acute brain slice preparation

Animals were deeply anesthetized with pentobarbital (50 mg/kg). After dissection, sagittal brain slices (250–300 µm) were prepared in chilled (4°C) dissection solution (in mM): 25.2 NaCl, 176 Sucrose, 2.5 KCl, 5 MgCl_2_, 1.2 CaCl_2_, 1.2 NaH_2_PO_4_, 10 Glucose, 26 NaHCO_3_, pH 7.4 bubbled with 95% O_2_/5% CO_2_. Slices were incubated for >1 hr in oxygenated artificial cerebrospinal fluid (aCSF) at room temperature (in mM): 125 NaCl, 2.5 KCl, 1 MgCl_2_, 2 CaCl_2_, 1.25 NaH_2_PO_4_, 10 Glucose, and 26 NaHCO_3_, pH 7.4. Slices were transferred to a chamber and continuously superfused (∼1 ml/min) with oxygenated aCSF at 32–34°C on the stage of an Olympus BX61 upright microscope equipped with a confocal microscopy (Fluoview 300) and 60× objective.

### Imaging and analysis of changes in capillary diameters, and U-46619 application

Vessels were loaded with 70 kDa Texas Red-dextran (TRD, Molecular Probes) through transcardial perfusion (200–400 µl at 12.5 mg/ml). Blood vessels were imaged >20 µm below the slice surface and identified as capillaries by their small diameter (<10 µm) and lack of smooth muscle cells. Blood vessels were visualized with confocal imaging using a 543 nm-laser. To measure vessel diameter over time, we determined the width of TRD fluorescence at every time-point of a time-lapse movie by drawing a line perpendicular to the vessel wall (line scan) and obtaining a reslice image using Image J. A custom-coded program (written by BL) in MATLAB (MathWorks, Inc.) automatically determined vessel diameter.

U-46619 was pressure applied (<3 psi) above the slice using a Picospritzer II (General Valve, NJ). All drugs and chemicals except those mentioned above were purchased from Sigma.

### LDF measurements, *in vivo* growth factors and EdU injection

Animals were anesthetized with intraperitoneal injection of 100 mg/kg ketamine-10 mg/kg xylazine and placed on a stereotaxic device (Kopf, Inc, RBM-1T). Scalp was retracted and two small burr holes (0.6 mm) were drilled for inserting the LDF microprobe (Oxford Optronics, UK) and a 33G needle with Hamilton syringe containing a drug and Cell Tracker Green. The probe diameter was 400 µm (two 200 µm-diameter glass fibers) and the centered inter-optode distance was 200 µm giving an estimated sampling volume of 0.02 µl. The probe was inserted near the SVZ (0.4/1.0 mm anterior to Bregma, 2.1/1.29 mm lateral to the midline, 3.0/2.1 mm ventral from the pia at a 7.1/8° angle for rats/mice) and the syringe into the lateral ventricle (0.4/1.0 mm, 2.75/1.53 mm, 5.4./3.59 mm at 52.9/42.4° angle for rats/mice). For cortical experiments, the respective probe and syringe coordinates were 1.0/1.0 mm posterior, 1.5/2.3 mm, and 0.8/0.9 mm at 30° angle. The animal vital signs were monitored with small animal plethysmograph on the footpad (MouseOx, Starr Life Sciences). Immediately following the experiments, the brains were removed and fixed in 4% paraformaldehyde to prepare serial sections and visualize penetration tracts and dye diffusion.

Recordings were continuous for 2 hours with three cycles of 1 µl U-46619 injection (Tocris) into the ventricle or a 30 min-injection of growth factors EGF and bFGF (PeproTech) at 0.5 mg/ml each and 10 µl/hour. EdU (50 mg/kg) intraperitoneal injections were given at the end of growth factor infusion, one hour prior to sacrifice.

### LDF analysis

The LDF signal was recorded and analyzed with Spike2 program (CED, Cambridge, UK). Control recordings prior to drug application were >30 min. The LDF signals were recorded at 50 Hz. The final time series were calculated as root mean square values of every 2 minute period. The percent changes were obtained by normalizing values to the baseline mean (2 min before drug injection) and the mean percent change was calculated from the last 5 min period of the record.

### Statistical analysis

Data were presented in Origin 8.0. Statistical significance was determined using the unpaired Student's t-test (p<0.05) in KyPlot 2.0. Data are presented as mean ± standard error of the mean (SEM).
